# A Scalable Microservices Architecture for Condition Monitoring and State-of-Health Tracking in Power Conversion Systems

**DOI:** 10.3390/s26041282

**Published:** 2026-02-16

**Authors:** José M. García-Campos, Abraham M. Alcaide, A. Letrado-Castellanos, Ramon Portillo, Jose I. Leon

**Affiliations:** 1 Department of Telematics Engineering, University of Sevilla, 41092 Sevilla, Spain; 2 Department of Electronic Engineering, University of Sevilla, 41092 Sevilla, Spain; 3 ENGREEN Laboratory of Engineering for Energy and Environmental Sustainability, University of Sevilla, 41092 Sevilla, Spain

**Keywords:** power converters, condition monitoring, predictive maintenance (PdM), microservices, scalability, Industry 4.0, Hardware-in-the-Loop, State-of-Health (SoH)

## Abstract

The role of power converters in modern electrical infrastructure (such as electric vehicle charging stations, battery energy storage systems and photovoltaic energy systems) has become critical. Given the high reliability required by these converters, continuous condition monitoring for predictive maintenance is mandatory. Traditional SCADA and HMI systems often face scalability bottlenecks and lack the flexibility in data aggregation and storage scalability required for long-term predictive maintenance. This paper proposes a scalable, containerized microservices-based architecture for degradation tracking and State-of-Health (SoH) monitoring in power conversion systems. The architecture features a decoupled four-layer structure, utilizing dedicated UDP servers for low-latency data ingestion, RabbitMQ (AMQP) for robust message routing, and a NoSQL (MongoDB) storage layer with a FastAPI interface. The proposed system was validated using a Hardware-in-the-Loop (HiL) setup with a Typhoon HIL606 simulator monitoring an Active Neutral Point Clamped (ANPC) power converter. Experimental stress tests demonstrated a Packet Delivery Ratio (PDR) of 1.0 at ingestion rates up to 100 messages per second (msgs/s) per node. The system exhibits transmission and processing overheads consistently below 5 ms, ensuring timely data availability for tracking thermal dynamics and parametric aging trends. This operational performance significantly exceeds the nominal requirement of 2 msgs/s for condition monitoring, ensuring robust data integrity. Finally, this modular approach provides the horizontal scalability necessary for Industry 4.0 integration, offering a high-performance framework for long-term health monitoring in modern power electronics.

## 1. Introduction

Societal energy demand is continuously growing with the massive adoption of electric vehicles, the proliferation of artificial intelligence agents and data-centers, among others. In this context, power converters are fundamental components in these modern electrical systems, serving as the interface for regulating and transferring power energy across a wide range of energy sources including renewable energy systems [1].

As demand grows for efficient power electronics systems, the reliability and availability of power converters become paramount [2]. Power converters are susceptible to several operational failures, making them critical factors for the operation of the entire system [3]. This becomes particularly important in some specific applications where the power converter availability factor is very high (above 99%), such as medical equipment or aerospace systems [4,5].

Numerous studies have identified power devices and capacitors as the most critical failure-prone components within power electronic systems [6]. The degradation of these devices can lead to system failures, raising critical safety concerns and incurring significant economic consequences. Thermo-mechanical stresses induced by thermal cycling are a major cause of these failures and greatly increase the likelihood of wear-out mechanisms such as bond-wire fatigue and solder degradation [7,8]. Conversely, in power capacitors, the operating temperature is directly correlated with their Remaining Useful Lifetime (RUL).

Traditional maintenance strategies relied on reliability data provided by manufacturers, such as those found in standard reference manuals. These models often assume constant failure rates, overlooking the wear-out mechanisms typically observed in power devices [9]. However, degrading phenomena are much more complex; consequently, these maintenance plans suffer from poor accuracy, often yielding unreliable RUL estimates.

Therefore, to improve maintenance plans, it is crucial to consider the impact of various stress factors such as temperature fluctuations that can accelerate degradation and lead to premature failures for power devices and capacitors [10]. To achieve this, key thermo-electrical magnitudes should be captured and post-processed to identify failure modes and degradation phenomena in critical components at the converter level.

Traditionally, Human–Machine Interface (HMI) and Supervisory Control and Data Acquisition (SCADA) systems have been the dominant frameworks for operating power conversion systems, owing to their maturity and widespread industrial adoption [11]. While these systems provide essential supervisory functions and long-term data persistence through dedicated logging systems, they are primarily optimized for steady-state, plant-level monitoring. Their architectures are inherently centralized and rely on polling-based communication cycles [12], which often employ aggressive data compression and downsampling to minimize storage overhead. Consequently, they are ill-suited for capturing the high-resolution, high-frequency transient electrical signatures required to analyze the complex wear-out mechanisms and State-of-Health (SoH) of power converters [13]. Furthermore, the increasing density of power units in modern distributed grids creates significant scalability bottlenecks. These centralized architectures struggle to manage concurrent high-throughput data streams without compromising data integrity or increasing latency [14,15]. This landscape is changing with the emergence of Industry 4.0, the Internet-of-Things (IoT), and edge-computing paradigms.

Industry 4.0 and IoT have become widely adopted paradigms for the development of monitoring systems in multiple application domains [16], including precision agriculture [17], healthcare [18], industrial environments [19], transportation [20], among others [21]. While these applications span diverse sectors, the need for real-time, intelligent monitoring has also gained prominence in the electrical domain [22], particularly as power systems become increasingly complex and data-driven. Consequently, supervisory and monitoring systems have become essential for electric power systems [23], with numerous studies highlighting their importance for energy-related platforms. Representative examples include IoT-based monitoring of photovoltaic facilities, focusing on the acquisition and visualization of key variables such as power, current, and irradiance [24], supervision and control of microgrid operation [25], and monitoring and fault detection in wind turbines, enabling both condition assessment [26] and predictive maintenance strategies [27].

As these systems expand in scale in size and complexity, the volume of data exchanged increases, imposing stricter requirements on data integrity. Therefore, the choice of application-layer communication protocol [28] (which governs how information is formatted, transported, and processed) must be carefully considered to optimize hardware resource usage, energy consumption, connection stability, and overall data reliability.

Common application-layer protocols in IoT monitoring ecosystems include MQTT (Message Queuing Telemetry Transport) [29], a lightweight publish/subscribe protocol suitable for low-power sensor networks and machine-to-machine communication; CoAP (Constrained Application Protocol) [30], designed for resource-constrained devices with efficient request/response communication over UDP; and AMQP (Advanced Message Queuing Protocol) [31], which provides robust message delivery, flexible routing, and reliable TCP-based transport, making it well-suited for complex and high-throughput systems.

Consequently, selecting an appropriate protocol is critical for modern power electronics monitoring systems, particularly where reliable and efficient data collection is paramount. While MQTT and CoAP offer lightweight solutions for resource-constrained devices, their message delivery guarantees and routing capabilities often prove insufficient in complex, high-throughput environments. In contrast, AMQP provides robust message delivery guarantees, flexible routing through exchanges and queues, and reliable TCP-based transport, thereby guaranteeing data integrity. Its support for both point-to-point and publish/subscribe models, combined with security features, makes AMQP especially suitable for supervisory and monitoring systems in power electronics, enabling real-time analysis, predictive maintenance, and accurate tracking of critical components.

However, selecting an efficient communication protocol addresses only the transport layer of the monitoring challenge. As the number of monitored assets increases, traditional monolithic software architectures—where data acquisition, processing, and storage are tightly coupled in a single executable—often become a bottleneck [32]. These centralized structures face limitations regarding horizontal scalability and often lack the flexibility to integrate heterogeneous devices or isolate faults effectively. Consequently, leveraging Industry 4.0 capabilities requires adopting a modular software architecture. A microservices-based approach, deployed via containerization, provides the decoupling to ensure that high-frequency data ingestion does not compromise the performance of data processing or storage performance, thereby facilitating true horizontal scalability in distributed power electronic systems [33].

Despite recent advancements in communication protocols, a significant gap remains between general purpose monitoring architectures and the stringent temporal resolution requirements of power electronics condition monitoring. This limitation becomes evident when analyzing the shortcomings of contemporary solutions:

- Next-Generation SCADA: Modern SCADA systems have evolved towards cloud connectivity, yet their core design remains optimized for supervisory control and steady-state data acquisition (typically 1 Hz to 0.1 Hz) [34]. They are ideal for grid stability management but lack the temporal resolution to capture the sub-millisecond electro-thermal dynamics required for assessing the SoH of power converters [35].
- Standard Edge IoT: While generic IoT platforms leverage protocols like MQTT or HTTP for broad interoperability, they are designed for discrete messaging rather than continuous data streaming. Relying on these TCP-based protocols for direct waveform acquisition introduces timing irregularities that challenge the capability to sustain gapless high-speed data acquisition. These solutions are well-suited for environmental monitoring but struggle to sustain the continuous streaming of high-frequency waveforms needed for transient fault diagnosis [36].

Therefore, this paper proposes a scalable, containerized microservices-based architecture designed specifically for the real-time monitoring and fault diagnosis of distributed power conversion systems. Unlike traditional centralized approaches, the proposed framework employs a decoupled four-layer structure to isolate data ingestion from processing and storage tasks. The system integrates a low-latency UDP-based ingestion layer with an AMQP message broker, the system ensures reliable high-frequency data acquisition while maintaining the flexibility required to integrate heterogeneous sensors and advanced diagnostic algorithms without altering the core infrastructure.

The main contributions of this work can be summarized as follows:

- A novel decentralized monitoring architecture: A modular software framework based on containerized microservices that overcomes the scalability bottlenecks of monolithic SCADA systems, allowing for the seamless integration of new power assets is proposed.
- High-frequency ingestion strategy: A specific implementation combining UDP for low-latency data transmission with RabbitMQ (AMQP) for reliable asynchronous buffering, ensuring zero data loss even under high-load conditions typical of power electronics switching frequencies is considered.
- Experimental characterization of system limits: A performance benchmark using synthetic software-emulated nodes to stress-test the architecture, analyzing the PDR and delay to define the maximum throughput boundaries and scalability saturation points prior to hardware integration has been conducted.
- Experimental validation via HiL: The proposed system is validated using a Typhoon HIL606 real-time simulator modeling an ANPC converter, demonstrating the system’s capability to handle ingestion rates up to 100 msgs/s per node with a Packet Delivery Ratio (PDR) of 1.0.

The remainder of this paper is organized as follows. Section 2 analyzes the current challenges in data observation and monitoring for power conversion systems, emphasizing the necessity of high-frequency data capture to accurately determine the SoH of critical components. Section 3 details the proposed modular software architecture. Section 4 presents an experimental characterization of the system’s performance limits using synthetic software-emulated nodes to evaluate horizontal scalability and resource saturation boundaries. Section 5 validates the complete architecture in a realistic HiL environment, demonstrating its operation with an ANPC converter under real-time conditions. Finally, Section 6 summarizes the main contributions and outlines future research directions.

## 2. Data Observation and Monitoring Issues

Studying the behavior of a physical system by observing its operational characteristics allows for proactive maintenance planning, which helps prevent significant deterioration or failure, a process known as condition monitoring (CM) [37]. As previously noted, power devices and capacitors in power converters are susceptible to failure due to thermal cycling (55% of cases), humidity or moisture (19%), contamination or dust (6%), and vibration (20%) [38]. These statistics underscore the importance of CM techniques for detecting early signs of degradation and scheduling maintenance accordingly.

**Figure 1:**
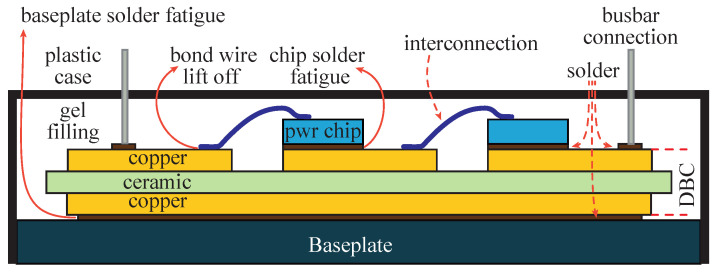
Cross-section of a conventional power module, and typical faults and their location.

**Figure 2:**
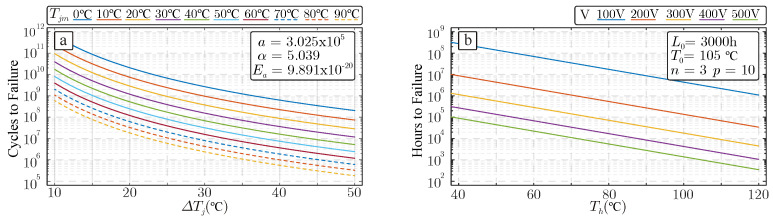
(**a**) Temperature cycle capability characteristic $N_{f}$ for a standard Semikron IGBT module. (**b**) Reliability model for electrolitic capacitor.

In power converters the most critical components are power devices and capacitors. In both cases, their operational temperature is a key factor. In Figure 1, a cross-section of a power device module is shown highlighting critical components such as bond wires. For power devices, there is a relationship between the number of thermal cycles of their junction temperature can undergo. This relationship is given by:

(1) $$\begin{array}{c} N_{f}=a\Delta T^{-\alpha }e^{\left(\frac{E_{a}}{k_{B}T_{jm}}\right)} \end{array}$$

where the coefficients *a*, α, and $E_{a}$ are obtained by experimental fitting; $k_{B}$ is the Boltzmann’s constant; ΔT is the thermal cycle amplitude (defined as the thermal swing); and $T_{jm}$ is the average junction temperature. These effects are illustrated in Figure 2, using a Semikron power electronics module as an example [39].

Directly measuring junction temperature in operational power devices within power converters is challenging; consequently, estimation techniques must be employed to monitor its behavior [40,41]. This estimation can be performed by using their conduction and switching loss to subsequently calculate its $T_{j}$ temperature by using Cauer or Foster thermal networks (see Figure 2b) [42,43]. Alternatively, $T_{j}$ temperature can be obtained by observing the evolution of several thermo-sensitive power device parameters [40,44].

Similarly, power capacitors’ lifetime is closely related to their operational hot-spot temperature (see Figure 2b). In this regard, the Capacitor’s RUL can be determined by:

(2) $$L_{c}=L_{0}{\left(\frac{V}{V_{0}}\right)}^{-n}2^{\frac{T_{0}-Th}{\rho }}$$

where parameters $L_{0}$, $T_{0}$ and $V_{0}$ are the nominal lifetime, temperature and voltage under the test conditions. Temperature $T_{h}$ is the hot-spot temperature of the device and coefficients *n* and ρ depend on the capacitor technology. For instance, for electrolytic capacitors *n* is defined in the range of [3–5] and ρ is equal to 10 [45,46].

In this case, $T_{h}$ temperature can be estimated by considering the $i_{RMS}$ thorough the device.

Therefore, it is clear that most relevant electrical magnitudes should be captured for further analysis and post-processing in order to determine the current SoH of the power converter and refining the maintenance scheduling of most critical components in power converters.

## 3. Proposed Architecture

### 3.1. System Overview

This work proposes a scalable, experiment-oriented architecture for the long-term condition monitoring and electrical analysis of power electronic converters. Built on a containerized microservices paradigm to ensure portability and fault isolation, the system is organized into four hierarchical layers:

1. Data acquisition and protocol adaptation: Interfaces directly with power converters to capture and packetize raw high-frequency data.
2. Message-oriented middleware: Acting as the communication backbone, this layer utilizes a message broker to decouple data producers from consumers.
3. Data processing and subscription services: Composed of independent subscriber microservices that consume data streams for processing and persistence, ensuring horizontal scalability.
4. Data storage and access interfaces: Manages the storage of heterogeneous data in a NoSQL database and provides a RESTful API for standardized retrieval and integration.

**Figure 3:**
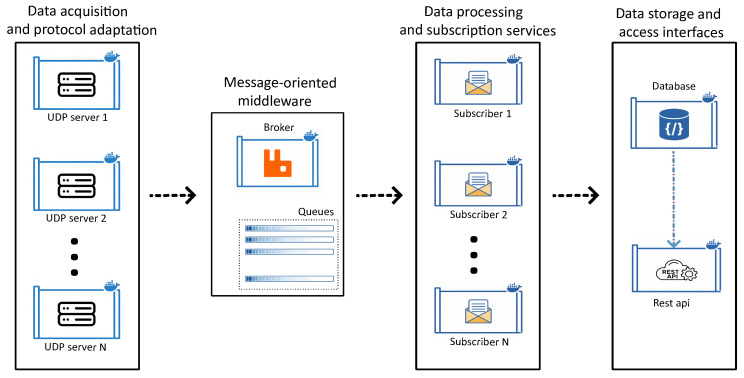
Proposed architecture scheme.

Figure 3 depicts the architecture’s conceptual diagram and information flow. This decoupled design ensures that each stage operates independently, facilitating efficient data processing and storage.

Each power converter under observation is treated as an independent data source, while experiments are defined as logical entities associated with a specific converter and operating condition. This abstraction enables the simultaneous execution of multiple experiments across different converters without introducing tight coupling between system components. Furthermore, a key feature of the proposed architecture is its ease of deployment, which is centralized through a single configuration file that registers all target converters and experiments; this approach minimizes manual setup and supports rapid scaling across multiple devices. To ensure reproducibility and facilitate the practical application of this architecture, the complete source code is publicly available in [47].

### 3.2. Data Acquisition and Protocol Adaptation Layer

This layer bridges the physical converters and the digital ecosystem, transforming raw high-frequency data for asynchronous processing. A dedicated, containerized UDP server is instantiated for each monitored converter [48]. The selection of UDP is based on the inherent capabilities of the converter hardware and for its minimal overhead and high throughput, which are critical for constrained micro-controllers in high-frequency telemetry [49]. To mitigate UDP’s lack of delivery guarantees, the layer implements an application-level verification process to ensure data consistency before propagation. While UDP is used here to meet specific timing constraints, the architecture remains protocol-agnostic, allowing the adoption of alternatives like TCP where hardware resources permit [50].

#### 3.2.1. Message Reception and Validation

**Figure 4:**
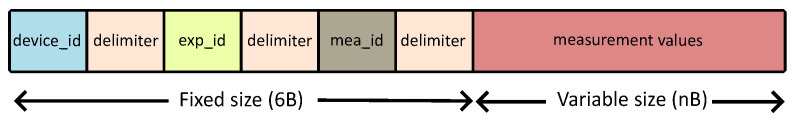
Raw message format.

Incoming messages are received as raw byte streams. Upon reception, the data undergo a sequence of processing steps to ensure structural integrity and semantic consistency before being forwarded to subsequent stages. Messages transmitted by the power converters are required to conform to the predefined format illustrated in Figure 4, which serves as the basis for the validation checks applied during reception.

The format illustrated in Figure 4 provides a general overview of the structure of the messages sent by the power converters. To better understand the composition of each message, Table 1 presents a detailed description of the individual fields, including their size and intended purpose.

Message reception involves the following validation steps:

(i) Message length validation: The total message length, in bytes, is verified against the expected structure (see Figure 4). The first part of the message is fixed at 6 bytes, with each field occupying 1 byte, while the measurement valuesfield is variable in size, depending on the type of measurement being performed. The validation ensures that the total length (see Equation (3)) equals 6 plus a multiple of 4 bytes, since the measurement values field consists of one or more values, each encoded as a 32-bit floating-point number
  (3) $$L_{\mathrm{total}}=L_{\mathrm{fixed}}+L_{\mathrm{variable}},\;\mathrm{with}\;L_{\mathrm{fixed}}=6\;\mathrm{bytes},\;L_{\mathrm{variable}}=4\cdot n,\;n\in N$$
  where *n* is the number of measurement values.
(ii) Message decoding: The byte stream is decoded into structured values by interpreting the fixed-size identifier fields (device_id, exp_id, mea_id and delimiter in Figure 4) as single-byte numerical values, while the measurement values (see Figure 4) field is decoded by converting each group of four bytes into a 32-bit floating-point number.
(iii) Measurement ID validation: The system defines a finite set of supported measurement values, representing all possible types of measurements that can be generated by the converters. The decoded measurement identifier (mea_id in Figure 4) is checked against this set.

This list should be updated according to the specific power converter topology and/or industry application. As an example, Appendix A lists all supported measurement types, representing the finite set of values that can be generated by the power converters and validated by the system. Notably, vector magnitudes length is a function of the fundamental frequency of the power system and the operating frequency of the converter. For instance a power converter connected to the grid (50 Hz) operated at 1 kHz generates vectors of 1000/50 = 20 elements. However, this length depends on the computational capabilities of the control platform of the power converter.

#### 3.2.2. Message Reconstruction and Broker Integration

**Figure 5:**
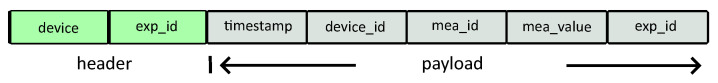
Reconstructed message format with header-payload structure.

Validated data is encapsulated into a structured header–payload message (see Figure 5) for middleware transmission. The header manages routing context, while the payload contains the measurement data defined in Table 1, appended with an acquisition timestamp.

### 3.3. Message-Oriented Middleware Layer

Serving as the asynchronous communication backbone, this layer is implemented using AMQP via a single containerized RabbitMQ broker. AMQP was chosen for its strong delivery guarantees and buffering, while RabbitMQ was selected for its maturity and proven performance. To ensure logical isolation, a dedicated headers exchange is assigned to each power converter, acting as the specific entry point for its generated data. Messages published to these exchanges—comprising header fields and payload (see Figure 5)—are routed to experiment-specific queues based on attributes such as the exp_id. This setup ensures logical isolation between converters, preventing cross-device data interference, and allows multiple experiments to run concurrently on the same converter while maintaining strict data separation.

Table 2 summarizes the key technical characteristics and configuration parameters of the middleware layer.

**Figure 6:**
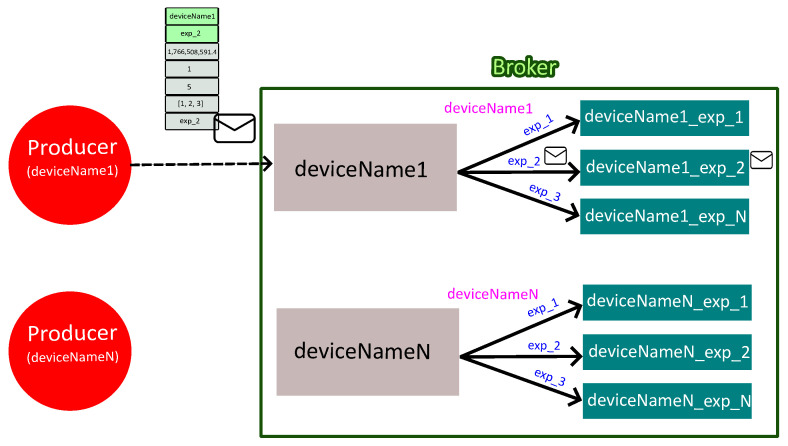
Information flow and routing within the message-oriented middleware.

To illustrate the operation of the message-oriented middleware layer, Figure 6 presents a representative example involving two power converters. In this example, each data acquisition service associated with a given converter (deviceName1 and deviceNameN in Figure 6) acts as a producer and publishes measurement data to the containerized RabbitMQ broker using AMQP.

Within the broker, each power converter is associated with its own headers exchange (e.g., deviceName1 or deviceNameN), which receives all messages originating from that device. Each message contains a payload with the measured values, among other data, and a set of header fields, including the power converter name and the experiment identifier. In the example illustrated in Figure 5, the message header fields take the values device = deviceName1 and exp_id = exp_2, which determines the routing of the message to the corresponding experiment-specific queue. Regarding the payload, the timestamp is 1,766,508,591.4, the device_id is 1, the mea_id is 5, the mea_value is [1, 2, 3]. Finally, the exp_id is exp_2. Eventually, the payload of the message will be stored in its corresponding queue (deviceName1_exp_2) until the appropriate consumer retrieves it.

### 3.4. Data Processing and Subscription Services

The processing layer consists of independent subscriber microservices, each deployed as an isolated container responsible for persisting data from a specific experiment’s queue of a given power converter. This one-to-one mapping ensures fault isolation and facilitates horizontal scaling. Data reliability is guaranteed through RabbitMQ acknowledgments, where messages are removed from the queue only after a confirmed database write.

Upon consumption, each subscriber executes a two-stage pipeline to guarantee data integrity:

1. Experiment confirmation: Messages are cross-referenced with the active experiment’s metadata based on the exp_id header. This secondary validation acts as a safeguard against routing errors, filtering out mismatched or malformed packets to ensure strict consistency.
2. Storage in database: Validated data is immediately written to the corresponding storage collection. This ensures near real-time persistence, preserving chronological integrity for subsequent retrieval.

**Figure 7:**
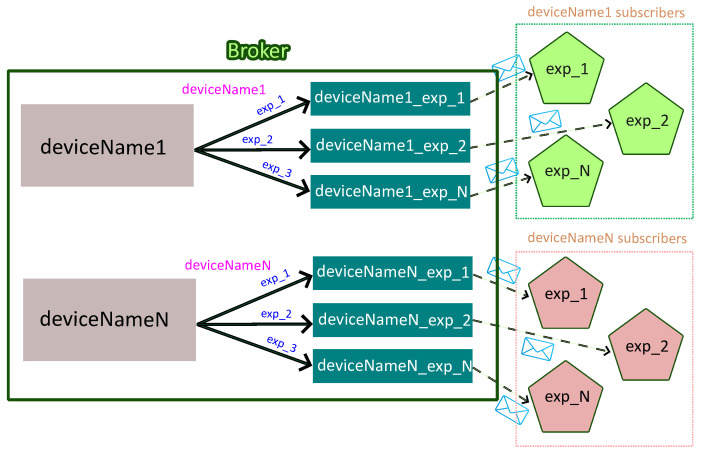
Architecture of the data processing and subscription layer.

Figure 7 depicts the specific data processing and subscription workflow described above. In this diagram, two distinct groups of subscribers can be identified: those corresponding to the queues of deviceName1 and those assigned to the queues of deviceNameN. As shown, the total number of subscriber instances, represented by pentagonal blocks in Figure 7, matches the number of active queues (six in this example).

### 3.5. Data Storage and Access Interfaces

This layer ensures long-term persistence and structured access to subscriber-processed measurements using a non-relational MongoDB database [51]. MongoDB was selected for its native support of complex document structures, avoiding the rigidity of relational schemas [52] and the inefficient data flattening required by standard TSDBs (e.g., InfluxDB) for high-dimensional vectors [53]. This enables the atomic storage of vectors, preserving the semantic integrity of heterogeneous datasets within a single record [54]. Comparative research on IoT workloads [55] validates this choice. While InfluxDB suffers from CPU spikes exceeding 600% under high concurrency, MongoDB demonstrates superior stability with moderate CPU growth (≈145%), ensuring scalable performance in containerized environments.

The database is structured by power converter, using distinct collections for each experiment to ensure isolation and scalability. To guarantee persistence, Docker volumes are employed to decouple the storage engine from the container lifecycle, preventing data loss during system updates or restarts. To maximize ingestion speed, subscribers perform direct write operations into MongoDB [56]. Bypassing intermediate layers minimizes communication overhead and prevents bottlenecks during high-frequency sampling.

A read-only RESTful API, built on the FastAPI framework [57], provides structured access to stored data. FastAPI was selected for its asynchronous execution, ensuring high performance under concurrent high-frequency requests [58]. The interface abstracts the underlying schema, delivering JSON payloads filtered by experiment, converter, or measurement identifiers. This interface also facilitates the integration of advanced diagnostic algorithms based on artificial intelligence or machine learning. By providing programmatic access to historical datasets, external AI agents can retrieve training data or perform offline validation of predictive maintenance models (e.g., RUL estimation) without interfering with the real-time data acquisition process. Furthermore, it facilitates aggregating data from multiple converters to train unsupervised learning models that identify irregular operating patterns across a fleet.

Security [59] is currently managed through private network isolation, suitable for a controlled laboratory, thus omitting public authentication. However, the architecture is fully compatible with industrial standards like OAuth2 and TLS [57], ensuring seamless adaptation for production environments where these protocols are mandatory.

Both the database and API operate as independent containerized services. This decoupling allows them to scale via replication in response to increased load without disrupting the upstream acquisition and processing layers.

### 3.6. System Deployment and Configuration

The deployment of the proposed monitoring system is designed to be modular and automated, ensuring the horizontal scalability and fault isolation principles discussed in Section 4.1. This process is managed by an orchestration layer by using a shell-based script that parses a centralized YAML configuration file to dynamically generate the infrastructure via Docker Compose.

The deployment workflow is executed in three sequential phases:

1. Network Infrastructure and Service Discovery: The orchestrator establishes a dedicated Docker bridge network to facilitate internal service discovery via logical hostnames, eliminating reliance on volatile static IPs.
2. Core Services Deployment: The script initializes the foundational, singleton components of the architecture: the MongoDB instance, the RabbitMQ message broker, and the RESTful API for data access. These services constitute the system’s backbone and are deployed using a static Docker Compose configuration.
3. Dynamic Service Instantiation and Specialization: For scalable components, the orchestrator performs dynamic instantiation using standardized Docker images. Individual container “specialization” is achieved via runtime parameter injection during the generation of specific Docker Compose files:
  - UDP servers: A unique container is instantiated per power converter. Shared images are parameterized with specific listening ports and converter identifiers, which determine the target RabbitMQ exchange for message publication.
  - Subscribers: Similarly, independent containers are instantiated per active experiment. Runtime parameters inject specific RabbitMQ routing details (exchange/queue), experiment identifiers, and MongoDB connection details (IP and port).

Finally, the orchestrator executes the docker-compose up command for all dynamically generated configurations. This automated approach minimizes manual intervention, mitigates configuration drift, and enables the system to scale from a single converter to a high-density experimental environment in seconds.

An example of this configuration file is shown in Appendix B.

## 4. Experimental Characterization of System Limits

The performance of the proposed monitoring architecture has been evaluated by characterizing its operational limits in terms of data reliability and delivery latency point of view. For this purpose, two primary metrics [60] are considered: the Packet Delivery Ratio (PDR), which measures the system’s capacity to reliably transport measurement data, and the delivery latency, defined as the time elapsed between the generation of a measurement and its successful persistence within storage layer.

The PDR figure of merit is defined as the ratio of successfully received messages to the total number of transmitted messages as shown in (4), expressed as a percentage:

(4) $$\mathrm{PDR}=\frac{N_{\mathrm{received}}}{N_{\mathrm{sent}}}\times 100\%$$

where $N_{\mathrm{received}}$ is the number of messages successfully received by the system, and $N_{\mathrm{sent}}$ is the total number of messages transmitted. In this context, a sent message refers to a message generated and transmitted by a (synthetic) power converter at a given point in time, whereas a received message is one that has been successfully stored in the database, ensuring it is available for subsequent processing and analysis.

Similarly, the architectural delay figure of merit is calculated as the time difference between the dispatch of the data packet and its final storage (5):

(5) $$\mathrm{delay}=t_{\mathrm{received}}-t_{\mathrm{sent}}$$

where $t_{\mathrm{sent}}$ and $t_{\mathrm{received}}$ represent the timestamps at which a message is sent and received, respectively. It is worth noting that this metric isolates the communication and processing overhead introduced by the proposed architecture, excluding the intrinsic physical acquisition window (≈20 ms for a 50 Hz fundamental cycle) required to construct the data vector.

The experimental analysis comprises two main phases. First, the maximum throughput that a single power converter can sustain without compromising data integrity or system responsiveness is determined (Section 4.1). Second, the system’s horizontal scalability is assessed by identifying the maximum number of concurrent converters that can be supported under high-load conditions (Section 4.2). These experiments yield insights into the architectural bottlenecks and offer practical guidelines for real-world deployments.

To validate the high efficiency and resource optimization of the proposed architecture, all experimental tests were conducted on a Raspberry Pi 4 Model B (4 GB RAM) running the Raspberry Pi OS operating system [61]. This hardware selection underscores the system’s lightweight design, demonstrating its ability to operate effectively on cost-efficient, resource-constrained edge computing devices without relying on high-performance server infrastructure.

Furthermore, it must be emphasized that the quantitative results presented are intrinsically linked to the specific hardware and software environment employed and should not be directly extrapolated to other architectures. While this study provides a clear baseline for the system’s behavior, a dedicated experimental characterization would be necessary for any alternative deployment to identify the specific operational boundaries and limitations of the corresponding architecture.

### 4.1. Impact of Message Rate on Reliability and Delay

Adhering to the methodology described above, this section evaluates the performance of a single monitoring node under varying ingestion rates. The primary objective is to determine the maximum throughput that the architecture can handle prior to compromising service quality in terms of reliability and latency.

To achieve this, the characterization is structured into five distinct experimental scenarios (see Table 3). Each experiment corresponds to a fixed message generation rate, from 1 to 10,000 messages per second (msg/s). To ensure statistical significance and account for potential outliers arising from inherent system and network variability, each scenario was independently repeated for 15 iterations. Each iteration lasted 10 min, providing a robust data sample to calculate consistent average values for both PDR and delay.

The PDR results (see Table 3) reveal a clear operational boundary for the proposed architecture on the selected hardware. While the system maintains perfect reliability (PDR = 1.0) for ingestion rates up to 100 msg/s (Experiments 1–3), a significant throughput saturation is observed as the rate scales further. In Experiment 4 (1000 msg/s), the PDR drops to 0.17, indicating that the system can no longer process the incoming message bursts in real-time, leading to buffer overflows or message rejection at the broker level. This trend is exacerbated in Experiment 5 (10,000 msg/s), where the PDR reaches 0.034. These results suggest that the effective processing capacity of a single-node Raspberry Pi 4 deployment peaks at approximately 340 messages per second under the current configuration.

The results for the delivery delay are summarized in Table 3. Remarkably, the system maintains a sub-5 millisecond delay across all experimental scenarios, with values ranging from 2.85 ms to 3.95 ms.

In the first three experiments (1 to 100 msg/s), the delay shows a slight downward trend, stabilizing at approximately 2.85 ms. This behavior is typical in message-oriented middleware, where the initial overhead of processing sporadic packets is amortized as the ingestion rate increases and the subscriber microservices reach a steady-state execution flow [62].

Importantly, these experiments utilize synthetic message streams rather than physical converters. Therefore, the results reflect the limits of the monitoring infrastructure—including the message broker, data processing, and storage layers—rather than the operational behavior of actual power converters. These findings provide practical guidance for defining operating parameters and identifying thresholds where system performance may degrade.

### 4.2. Scalability Analysis with Multiple Simulated Converters

Building upon the performance characterization established in the previous section, this subsection evaluates the horizontal scalability of the proposed architecture. The primary objective is to determine the operational limits of the system when transitioning from a single-node setup to a multi-device environment, thereby assessing its suitability for large-scale power conversion plants. Following the findings from Section 4.1, which identified 100 msg/s as the stability threshold for the current hardware configuration, this experimental phase maintains a constant aggregate throughput of 100 msg/s.

This workload is distributed across an increasing number of synthetic power converter instances (*N*). It is important to emphasize that these nodes do not consist of physical hardware; instead, they are synthetic entities emulated via software. This approach allows for an isolated assessment of the impact of concurrency and resource orchestration on system stability, ensuring that the performance evaluation focuses strictly on the architecture’s ability to manage multiple simultaneous data streams.

The experiments are defined as follows:

To ensure statistical reliability and maintain consistency with the methodology established in Section 4.1, each experimental configuration was conducted over 15 iterations. The results obtained from this scalability characterization are summarized in Table 4, which present the average PDR and delivery delay for each experiment.

These results reveal that the architecture maintains its operational integrity up to a limit of N = 4 concurrent devices. While for N ≤4 a PDR of 1.0 and delays below 9 ms are guaranteed, from N = 5 onwards, a significant degradation occurs, with the PDR dropping to 0.92 and the delay increasing non-linearly to 24.85 ms. This behavior identifies N = 4 as the saturation threshold for the evaluated hardware, where instability does not derive from the total data volume—maintained constant at 100 msg/s—but from the overhead of managing multiple independent network streams and the resource contention among containerized instances for the system’s physical resources.

### 4.3. Implications for System Deployment

The experimental characterization of the proposed architecture reveals a significant operational margin that is critical for industrial power conversion applications. The stress tests identified a maximum stable throughput of 100 msg/s per monitoring node before reaching saturation. However, the specific requirements for condition monitoring, focused on tracking slow-evolving degradation variables like thermal stress or electrolyte loss, are typically satisfied with a sampling rate of 2 msg/s per power converter.

Consequently, under nominal operating conditions, a single converter utilizes only 2% of the node’s verified communication capacity. This extensive headroom is a deliberate architectural advantage that ensures two critical deployment capabilities:

1. Vertical Scalability and Reliability: Operating at a fraction of its total capacity allows a single low-cost edge device (e.g., Raspberry Pi 4) to concurrently monitor multiple power converters—theoretically up to 50 units at the target rate—without compromising stability. Furthermore, this 98% safety margin provides the necessary resilience to handle unpredictable data bursts or high-priority traffic during transient fault events, preventing packet loss and ensuring deterministic latency in critical situations.
2. Edge Computing Readiness: By maintaining a lightweight communication overhead, the system preserves the majority of the node’s computational resources (CPU and memory) for the execution of the diagnostic layer. This enables the implementation of real-time health-state estimation algorithms, such as Fast Fourier Transform (FFT) analysis or machine learning-based classification, directly at the edge.

## 5. Use Case—Hardware-in-the-Loop Active Neutral Point Clamped Power Converter Real Time Simulation

To evaluate the proposed architecture under realistic conditions, this section presents a validation case study centered on the monitoring of a grid-tied Photovoltaic (PV) Power Plant. The aims is to validate the system’s ability to acquire, process, and store high-frequency telemetry data from complex power electronics system without disrupting ongoing operations.

**Figure 8:**
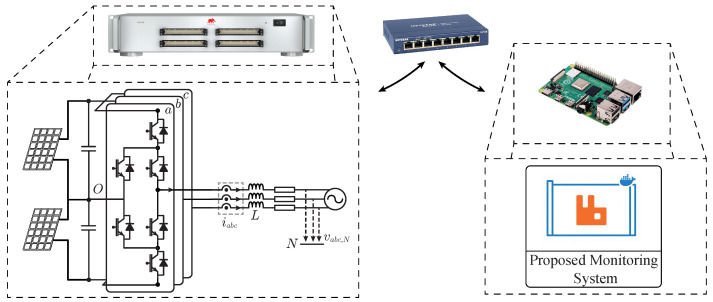
Experimental setup considering HIL system and the proposed monitoring system.

The monitoring architecture was validated using a Hardware-in-the-Loop (HiL) real-time simulator, specifically the HIL606 from Typhoon. Consistent with the characterization presented in Section 4, the acquisition system was deployed on a Raspberry Pi 4 Model B (4GB RAM) to replicate a resource-constrained edge environment. In this scenario, the HIL device emulates an Active Neutral Point Clamped (ANPC) converter serving as the electrical interface for the PV plant. An scheme of the system is shown in Figure 8 where the emulated power system architecture based on the ANPC power converter is also drawn.

The experimental setup is established within a controlled laboratory environment. Concerning the network topology, both the Typhoon HIL606 and the Raspberry Pi are connected to the same dedicated Local Area Network (LAN) via Gigabit Ethernet switch device. This configuration enables the data acquisition platform operates in an air-gapped mode (i.e., isolated from the Internet), thereby preserving high-frequency telemetry integrity and preventing packet loss due to external network congestion. This aligns with standard practices in power electronic systems, where converters remain isolated from the Internet for safety, restricting external connectivity to administrative purposes.

In this validation scenario, the Typhoon HIL simulator emulates the ANPC converter following a pre-defined mission profile (that means, imposing active and reactive power references ($P^{★},Q^{★}$) for a PV application. The control scheme used in this scenario follows the conventional dq synchronous-frame based on linear controllers for the sake of simplicity and the modulation technique is based on conventional level-shifted pulse width modulation (LS-PWM). After starting up the system, the HIL606 is streaming critical telemetry data to the proposed acquisition platform. The data export adheres to the classification criterion reported in Table A1, encompassing measurements such as three-phase grid voltages, phase currents, and estimated IGBT conduction losses. Specifically, the transmission strategy follows a multi-rate approach: messages in the catalog from IDs 1 to 6 (corresponding to internal vector magnitudes) are dispatched periodically every 5 s, assuming a steady state (sending a vector containing information for one single period), whereas messages from IDs 10 to 15 (containing average or RMS metrics) are transmitted at 1 s intervals. This injection profile, which interleaves instantaneous values for transient analysis with downsampled metrics for steady-state supervision, effectively emulates the complex and heterogeneous data traffic patterns typical of real-world industrial converters.

**Figure 9:**
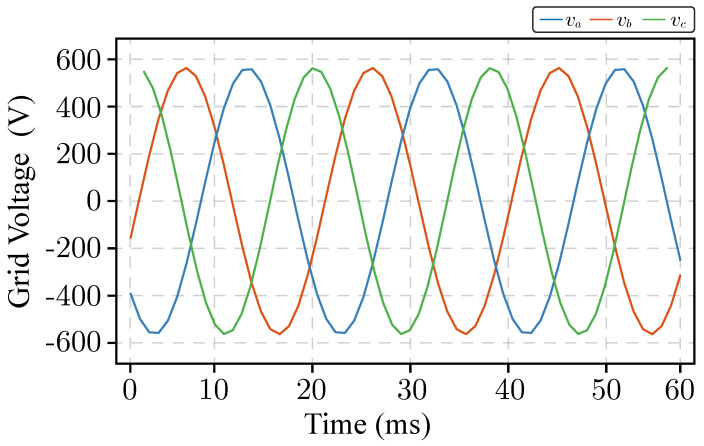
Grid voltage captured in the Typhoon HIL system and sent to the monitoring system.

As a result of the HIL606 operation, some data have been captured in the proposed monitoring system from emulated power converter and lately recovered from the database for presentation purposes in this manuscript. An example of internal data vector capturing is shown in Figure 9 where the grid voltage is shown.

**Figure 10:**
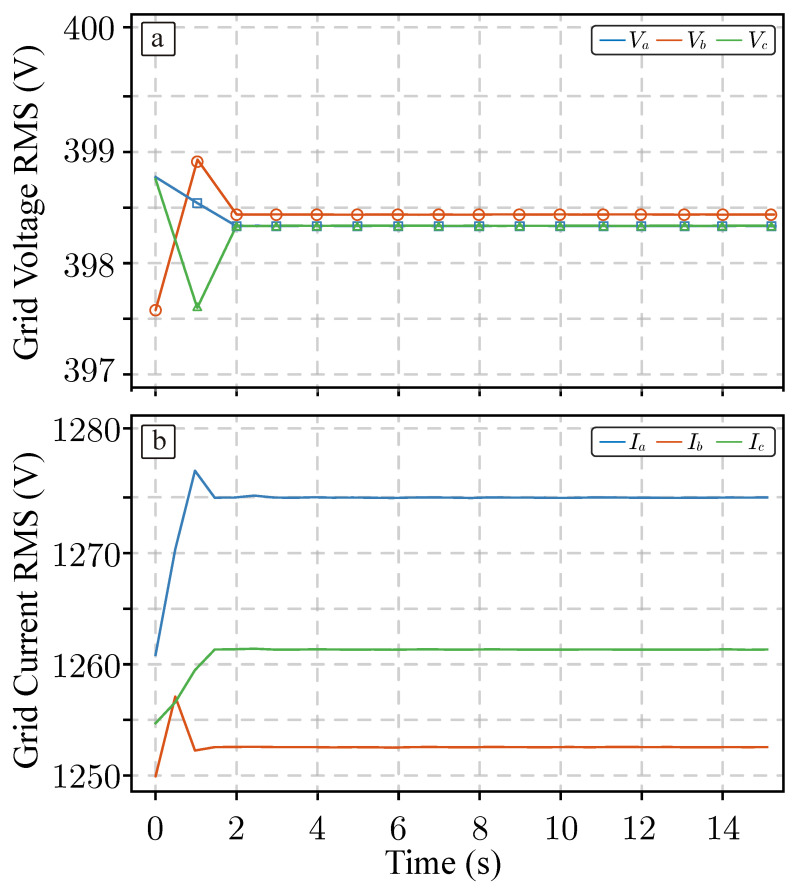
Magnitudes capture in the Typhoon system and sent to the monitoring system: (**a**) grid voltage RMS; (**b**) output current.

**Figure 11:**
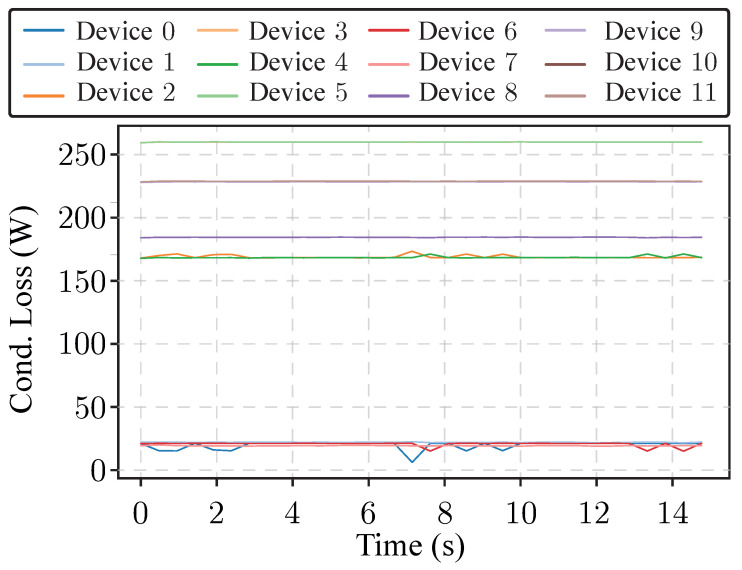
Conduction losses of the power device presents in phase a captured by the Typhoon HIL system and sent to the monitoring system.

As mentioned, the datalogging process has performed using a multi-rate process. To demonstrate this fact, RMS values of significant magnitudes have been capturing by message ID 10, 11 and 13 during a portion of time equals to 15 s. These trends are represented in Figure 10 and Figure 11, respectively. This feature is specially important to determine if the power system is correctly operated. In the case of conduction loss of the power devices of the phase a are internally sampled and forwarded to the monitoring system for later processing. It is worth to be mentioned the amount of transmitted data, specially for message ID = 13 where the average conduction loss of 12 devices are logged without data-loss, an example of the capture for data vector is shown. This functionality is very helpful for early fault detection and/or RUL observation because it is possible to capture correlated data within the power converter.

Listing 1 shows a raw data fragment from the MongoDB collection, illustrating how messages with different measurement IDs (id_mea) and vector lengths (mea_value) are chronologically persisted as they arrive from the microservices network.

**Listing 1.** Representative subset of data persisted in MongoDB, illustrating the interleaving of messages with varying measurement IDs and vector lengths.
{

 "_id": "695437fceca9cc949c9ec03a",

 "timestamp": 1767127033.4299982,

 "device": "typhoon",

 "id_mea": 11,

 "mea_value": Array (3) [

    0: 1251.0855712890625,

    1: 1261.38623046875,

    2: 1255.5899658203125

 ],

 "experiment_id": "experiment_3"

}

{

 "_id": "695437fceca9cc949c9ec03c",

 "timestamp": 1767127033.4384139,

 "device": "typhoon",

 "id_mea": 13,

 "mea_value": Array (12) [

    0: 21.117103576660156,

    1: 22.158349990844727,

    2: 167.96240234375,

    …

    11: 228.3522186279297

 ],

 "experiment_id": "experiment_3"

}

{

 "_id": "695437fceca9cc949c9ec03e",

 "timestamp": 1767127033.446452,

 "device": "typhoon",

 "id_mea": 1,

 "mea_value": Array (180) [

    0: -154.28805541992188,

    1: 21.586299896240234,

    …

    179: 562.0804443359375

 ],

 "experiment_id": "experiment_3"

}


The experimental results confirm that the proposed architecture, deployed on a standard Raspberry Pi 4, successfully handles the dual requirement of high-frequency waveform reconstruction and long-term trend logging. The system maintained data integrity under the emulated traffic load, demonstrating that a decoupled microservices approach is a viable and cost-effective solution for the real-time monitoring of power conversion assets.

## 6. Conclusions and Future Works

This study presents a scalable, containerized monitoring architecture designed to address the rigidity and throughput limitations of traditional SCADA systems in power electronics. By employing a decoupled four-layer framework based on independent microservices, the proposed solution supports the high-frequency telemetry ingestion while maintaining system stability and data integrity.

The validation process was conducted in two complementary phases. First, synthetic characterization established the system’s maximum throughput boundaries. Subsequently, and most importantly, the architecture was assessed within a representative environment using a Hardware-in-the-Loop (HiL) setup. In this environment, a Typhoon HIL606 simulator emulated a grid-tied Photovoltaic Power Plant driven by a standard mission profile, replicating the dynamics of an ANPC converter in real-time.

Experimental results in representative scenario demonstrated that the system, deployed on a resource-constrained Raspberry Pi 4, effectively processes heterogeneous traffic patterns-combining instantaneous waveforms for fault diagnosis and RMS metrics for steady-state supervision. This versatility is facilitated by the non-relational (NoSQL) nature of the storage layer, which enables the system to ingest and persist diverse data structures independent of rigid schema limitations. Furthermore, employing containerization was instrumental in this deployment, providing the modularity necessary to support horizontal scalability and efficient resource orchestration at the edge. The architecture maintained a PDR of 1.0 at ingestion rates of up to 100 msgs/s per node, demonstrating the feasibility of implementing reliable monitoring using cost-effective hardware. To extend this scalability to massive deployments, the architecture is designed to evolve towards container orchestration platforms such as Kubernetes, enabling automated scaling, self-healing capabilities, and the centralized lifecycle management of the microservices fleet across extensive industrial clusters.

Building upon the successful HIL validation, future research targets the deployment on physical ANPC converters to assess the architecture’s robustness under real-world electromagnetic interference and thermal constraints. To bridge the gap towards industrial adoption, the software framework will be enhanced by integrating digital twin interfaces and RUL estimation algorithms directly into dedicated containers. This enables real-time synchronization between the physical asset and its virtual model, facilitating predictive maintenance strategies to minimize unplanned downtime. Furthermore, the system’s scalability will be extended to a hybrid edge cloud topology aimed at the centralized supervision of distributed energy resources, such as PV inverters, battery energy storage systems, and EV charging stations, allowing for the unified observability of extensive device fleets. Finally, to ensure compatibility with brownfield infrastructure, future microservices will bridge internal telemetry with standard industrial protocols such as IEC 61850 or Modbus TCP, facilitating seamless integration with existing SCADA monitoring systems.

## Figures and Tables

**Table 1:** Raw message.

Field	Size (Bytes)	Description
device_id	1	Power converter identifier
exp_id	1	Identifier of the experiment
mea_id	1	Identifier of the measured quantity
measurement values	4xn	Values of the measurement
delimiter	1	Field separator

**Table 2:** Key characteristics of the message-oriented middleware layer.

Feature	Description	Purpose
Protocol	AMQP	Standardized communication
Broker	RabbitMQ	AMQP implementation
Exchange	Headers	Header-based routing
Bindings	Based on exp_id and device	Experiment and device separation
Scalability	Horizontal	System expansion

**Table 3:** Message rate—features and results.

Experiment	Messages/s	PDR	Delay (ms)
1	1	1.0	3.95
2	10	1.0	3.58
3	100	1.0	2.85
4	1000	0.174	3.05
5	10,000	0.034	3.27

**Table 4:** Scalability—features and results.

Experiment	Device Number	PDR	Delay (ms)
1	2	1.00	3.14
2	4	1.00	8.93
3	5	0.92	24.85
4	6	0.78	27.65

## Data Availability

The raw data supporting the conclusions of this article will be made available by the authors on request.

## Appendix A. Measurement Data Dictionary

Table A1 provides the complete mapping of variable identifiers (IDs) to physical quantities for the 3L ANPC converter implementation. These identifiers are utilized by the UDP serialization logic and the MongoDB document structure to categorize incoming telemetry.

**Table A1:** Supported measurement types for a 3L ANPC Converter operated in a grid connection application and a 1 kHz control frequency.

ID	Description
1	Instantaneous three-phase voltages in vector format, 20 components
2	Instantaneous three-phase currents in vector format, 20 components
3	Instantaneous values of DC, voltages of the 2 capacitors, and input current to the converter, 20 components
4	Instantaneous conduction losses in the devices, 20 components each
5	Instantaneous switching losses in the devices, 20 components each
6	Instantaneous junction temperatures of the devices, 20 components each
10	RMS value of three-phase voltages
11	RMS value of three-phase currents
12	RMS value of the converter DC, voltages of the 2 capacitors, and input current to the converter
13	RMS value of conduction power losses in the devices
14	RMS value of switching power losses in the devices
15	RMS value of junction temperatures in the devices

## Appendix B. Architectural Deployment

The deployment automation, as detailed in Section 3.6, is driven by a centralized configuration file that defines the system topology and service parameters. Listing A1 presents the standard YAML template used as input for the orchestration script, with its key components detailed below:

- network: Defines the container network to which all containers (API, database, broker and UDP servers) are attached, enabling inter-container communication.
- api: Specifies the IP address for the RESTful API. Only one API container is created, independent of the number of monitored converters.
- services: Lists all power converters to be monitored. For each device:
  - device: Human-readable name of the converter.
  - id: Unique numerical identifier used by the UDP server to route messages to the corresponding broker exchange.
  - port: UDP port for incoming data. Each converter has a dedicated UDP server container.
  - ipbroker: IP of the central RabbitMQ broker.
  - experiments: List of experiments for the device, creating dedicated queues in the broker and subscriber services.

**Listing A1.** YAML template for architectural deployment.
network: name: container_network api: ip: "API_IP" services: - device: deviceName1 id: 1 port: UDP_port_1 ipbroker: "Broker_IP" experiments: - exp_1 - exp_2 - exp_N - device: deviceNameN id: 2 port: UDP_port_2 ipbroker: "Broker_IP" experiments: - exp_1 - exp_2 - exp_N
